# Antroquinonol Lowers Brain Amyloid-β Levels and Improves Spatial Learning and Memory in a Transgenic Mouse Model of Alzheimer’s Disease

**DOI:** 10.1038/srep15067

**Published:** 2015-10-15

**Authors:** Wen-Han Chang, Miles C. Chen, Irene H. Cheng

**Affiliations:** 1 Institute of Brain Science, National Yang-Ming University, Taipei, Taiwan; 2 Division of Biological Chemistry, R&D, Golden Biotechnology Corporation, New Taipei City, Taiwan; 3 Brain Research Center, National Yang-Ming University, Taipei, Taiwan; 4 Infection and Immunity Research Center, National Yang-Ming University, Taipei, Taiwan

## Abstract

Alzheimer’s disease (AD) is the most common form of dementia. The deposition of brain amyloid-β peptides (Aβ), which are cleaved from amyloid precursor protein (APP), is one of the pathological hallmarks of AD. Aβ-induced oxidative stress and neuroinflammation play important roles in the pathogenesis of AD. Antroquinonol, a ubiquinone derivative isolated from *Antrodia camphorata*, has been shown to reduce oxidative stress and inflammatory cytokines via activating the nuclear transcription factor erythroid-2-related factor 2 (Nrf2) pathway, which is downregulated in AD. Therefore, we examined whether antroquinonol could improve AD-like pathological and behavioral deficits in the *APP* transgenic mouse model. We found that antroquinonol was able to cross the blood-brain barrier and had no adverse effects via oral intake. Two months of antroquinonol consumption improved learning and memory in the Morris water maze test, reduced hippocampal Aβ levels, and reduced the degree of astrogliosis. These effects may be mediated through the increase of Nrf2 and the decrease of histone deacetylase 2 (HDAC2) levels. These findings suggest that antroquinonol could have beneficial effects on AD-like deficits in *APP* transgenic mouse.

Alzheimer’s disease (AD), the most common form of dementia, affects millions of people every year. Unfortunately, to date, there is no effective treatment for the disease. Abnormal accumulation of extracellular amyloid-β peptides (Aβ) in amyloid plaques is one of the pathological hallmarks in the brain of AD patients. Aβ peptides containing 40 (Aβ40) or 42 (Aβ42) amino acids are cleaved from amyloid precursor protein (APP) by β- and γ-secretases^1^. The importance of Aβ in the etiology of AD has been demonstrated in many *in vivo* and *in vitro* systems. Multiple transgenic mouse lines containing familial AD mutations in APP have been generated, which develop amyloid plaques in their brains and show impairments in learning and memory^2^,^3^,^4^. Reducing levels of Aβ through genetic or pharmacological approaches in these models is often linked to the alleviation of their cognitive impairments^5^,^6^.

Inflammation and oxidative stress are two of the major factors resulting in neurodegeneration during AD pathogenesis^7^. Aβ-induced astrocyte activation is involved in the production of proinflammatory cytokines and reactive oxygen species which contribute to synaptic loss and memory decline^8^. Astrocytes are immune-like cells that become reactive in response to neuronal injury. Astrogliosis has been commonly observed in AD patients^9^ and mouse models^10^,^11^. Astrogliosis usually leads to the production of cytokines and reactive oxygen species, thereby triggering inflammation and oxidative stress. Increased lipid peroxidation as well as protein and DNA oxidation are found in AD brains^12^, AD cerebrospinal fluid^13^ and neurons derived from AD patients^14^. Antioxidant treatments in the early stages of pathogenesis were able to alleviate the functional impairment^15^,^16^,^17^,^18^ and to reduce brain Aβ in AD mouse models^17^,^19^,^20^.

Signaling via nuclear transcription factor erythroid-2-related factor 2 (Nrf2), a transcription factor regulating anti-oxidative genes, is attenuated in AD patients^21^ and mouse models^22^. Activation of Nrf2 signaling is essential for counteracting the oxidative damage and Aβ-induced toxicity^23^. Antroquinonol, an active compound purified from the polyporus mushroom *Antrodia camphorata*, has antioxidative and anti-inflammatory effects^24^,^25^,^26^. *Antrodia camphorata* has been passed safe for human use in the clinical trial (ClinicalTrials.gov Identifier:NCT01007656) and is commonly used as an herbal remedy for cancer, hypertension, and hangover. In this study, we explored whether antroquinonol treatment can ameliorate the AD-like phenotype seen in *APP* transgenic mouse.

## Results

### Biosafety of antroquinonol administration

**Figure 1:**
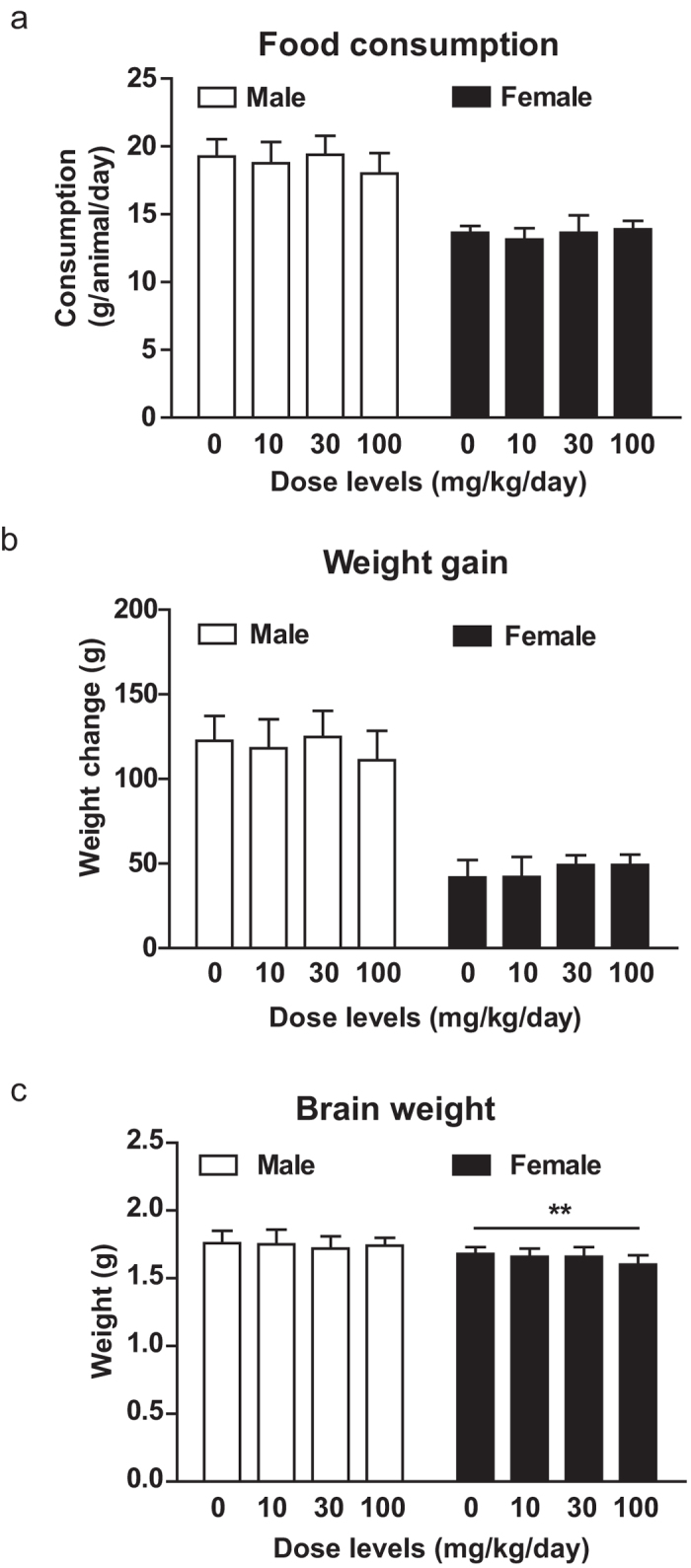
Biosafety test of antroquinonol. SD rats were divided into four groups receiving 0, 10, 30, and 100 mg/kg/day of antroquinonol by gavage for 4 weeks. (**a**) Food consumption, (**b**) body weight gain were recorded weekly and (**c**) brain weight was analyzed after 4 weeks’ administration. (**a**,**b**) Neither male nor female rats showed any significant changes in food consumption or body weight gain after the treatment of antroquinonol, compared to vehicle. (**c**) Consumption of 10, and 30 mg/kg/day of antroquinonol did not significantly alter brain weight, compared to control diet. However, consumption of 100 mg/kg/day of antroquinonol reduced brain weight in female rats. Data are presented as mean ± SD. Results were analyzed by ANOVA. Each group comprises 10 males and 10 females.

Before examining the efficacy of antroquinonol in AD model, the biosafety of antroquinonol was examined by repeated administration of 10, 30, and 100 mg/kg/day of antroquinonol, or vehicle, to Sprague-Dawley (SD) rats for 4 weeks. These rats had no significant changes in food consumption, loss of body weight, or most organ weights among these groups (Fig. 1 and Supplementary Table 1). However, females administered 100 mg/kg/day of antroquinonol had a lower brain weight than vehicle control group (Fig. 1c). In addition, there were changes in the weights of liver, thymus, and adrenal gland in animals receiving 100 mg/kg/day of antroquinonol (Supplementary Table 1). Animals receiving 30 mg/kg/day or lower showed no obvious detriments in clinical condition, ophthalmoscopy, food consumption, hematology, blood chemistry, urinalysis, gross pathology, or histopathology. Animals receiving 100 mg/kg/day showed some histopathological changes (Supplementary Table 2), and some clinical signs occurred sporadically, including firmness of the abdomen, loose feces, and predose salivation. To sum up, antroquinonol dosages below 30 mg/kg/day do not appear to be associated with any adverse effects.

### Blood-brain barrier penetration of antroquinonol

**Figure 2:**
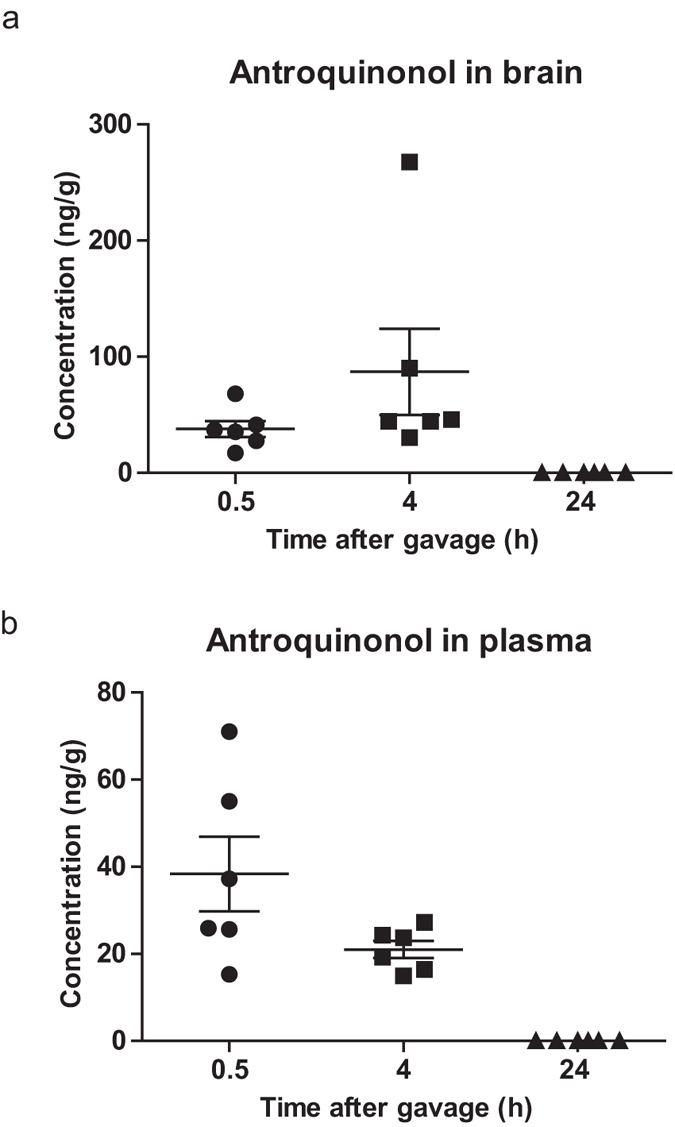
Antroquinonol could cross the blood-brain barrier. Mice were administered a single dose of 30 mg/kg of antroquinonol orally, and a necropsy was conducted at 0.5, 4, or 24 hours after administration. Antroquinonol levels were detected in brain (**a**) and plasma (**b**) within 4 hours of administration. Antroquinonol levels were not detectable 24 hours after administration. Data are presented as mean ± SEM. *n* = 6 for each time point.

To assess the bioavailability and blood-brain barrier penetration of antroquinonol, mice were orally administered 30 mg/kg of antroquinonol. Tissue and plasma were collected 0.5, 4, or 24 hours after gavage, and their antroquinonol concentration were determined by liquid chromatography-tandem mass spectrometry (LC-MS/MS). After 0.5 hours and 4 hours, a 30 mg/kg oral dose of antroquinonol yielded a plasma concentration of 38.34 ng/g and 20.98 ng/g, and a brain concentration of 37.75 ng/g and 87.02 ng/g (Fig. 2). After 24 hours, the concentrations of antroquinonol in both plasma and brain were returned to undetectable level. Antroquinonol was also detected in many other tissues such as the spleen, stomach, and liver (Supplementary Table 3). These results indicate that antroquinonol has good bioavailability and can cross the blood-brain barrier at the dosage without adverse effect.

### Antroquinonol consumption on general behaviors in APP transgenic mice

**Figure 3:**
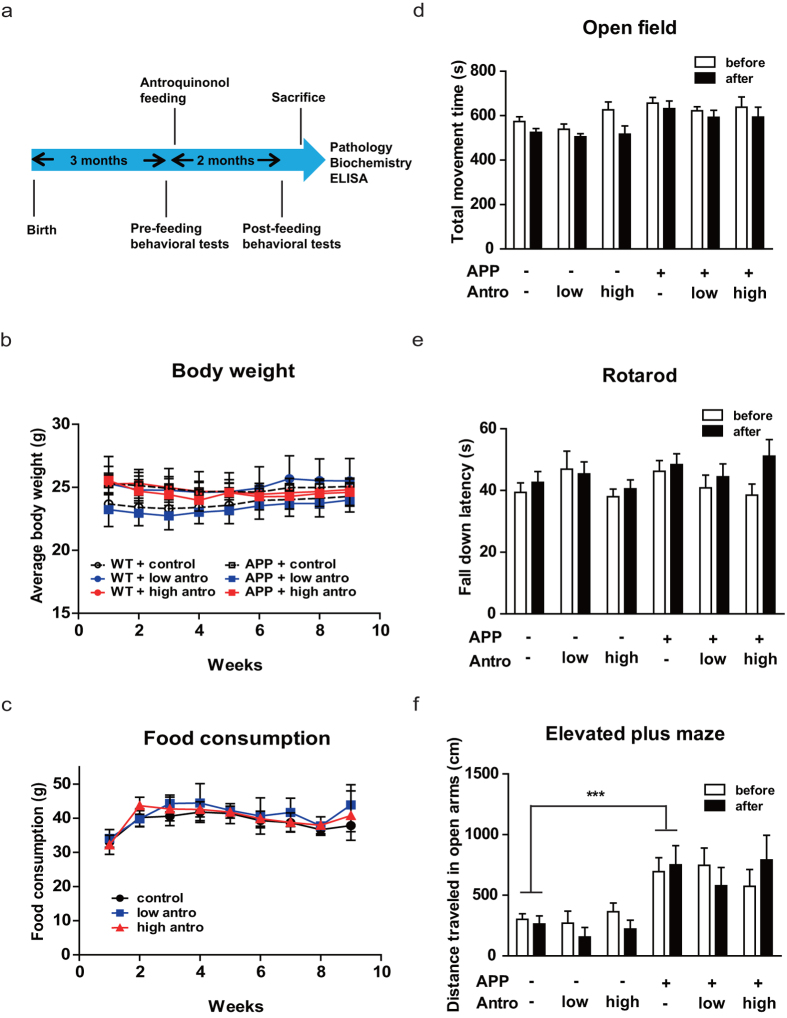
Antroquinonol (Antro) did not change body weight and general behaviors in WT and *APP* mice. (**a**) Experimental design: Starting at 3 months of age, WT and *APP* mice were given low (7.2 mg/kg/day) and high (34.2 mg/kg/day) doses of antroquinonol for 2 months. Open field, rotarod, and elevated plus maze tests were performed before and after treatment. The water maze test was performed after 2 months of antroquinonol consumption. Pathological and biochemical examinations were performed after all the mice were sacrificed. (**b**,**c**) The body weight change (**b**) and food consumption (**c**) were recorded weekly during the 2-month treatment period. (**d**) In the open field test, WT and *APP* mice showed similar movement times before and after antroquinonol consumption. (**e**) In the rotarod test, WT and *APP* mice had similar fall-down latencies. (**f**) In the elevated plus maze test, *APP* mice travelled a longer distance in the open arms than WT mice, and antroquinonol consumption did not change this. Data are presented as mean ± SEM. Results were analyzed by repeated measures ANOVA (**b**,**c**) and two-way ANOVA (**d**–**f**). Number of mice: WT + control = 15, WT + low antro = 6, WT + high antro = 8, APP + control = 11, APP + low antro = 8, APP + high antro = 6. ***P < 0.001 vs. WT + control.

We used *APP* transgenic mouse as our AD animal model to examine whether antroquinonol could ameliorate AD-like deficits *in vivo*. The *APP* mouse line (J20) used in this study develop spatial memory impairments at 4 months of age^27^, so all treatments were started at 3 months of age for 2 months (Fig. 3a). To determine the dosage effects, we fed mice diet containing 0% (control), 0.003% (low dose), and 0.015% (high dose) antroquinonol. After measuring the weight of diet intake, each mouse on average consumed 7.2 mg/kg/day (low dose) or 34.2 mg/kg/day (high dose) of antroquinonol, roughly equivalent to an adult human consuming 0.58 mg/kg/day or 2.77 mg/kg/day^28^. The average body weight and food consumption were not significantly altered during the 2 months of antroquinonol treatment (Fig. 3b,c).

Before and after antroquinonol consumption, we used the open field test to monitor locomotor activity, the elevated plus maze to screen for anxiety-related behavior, and the rotarod test to examine the motor co-ordination of these mice (Fig. 3d–f). There were no significant changes in total locomotor activity (Fig. 3d) or motor co-ordination (Fig. 3e) among all groups. In the elevated plus maze test, *APP* mice travelled a longer distance in the open arms compared to wild-type (WT) mice, as reported previously^4^ (Fig. 3f), but antroquinonol did not significantly alter this anxiety behavior within the groups.

### Improvement in spatial learning and memory in APP transgenic mice by antroquinonol

**Figure 4:**
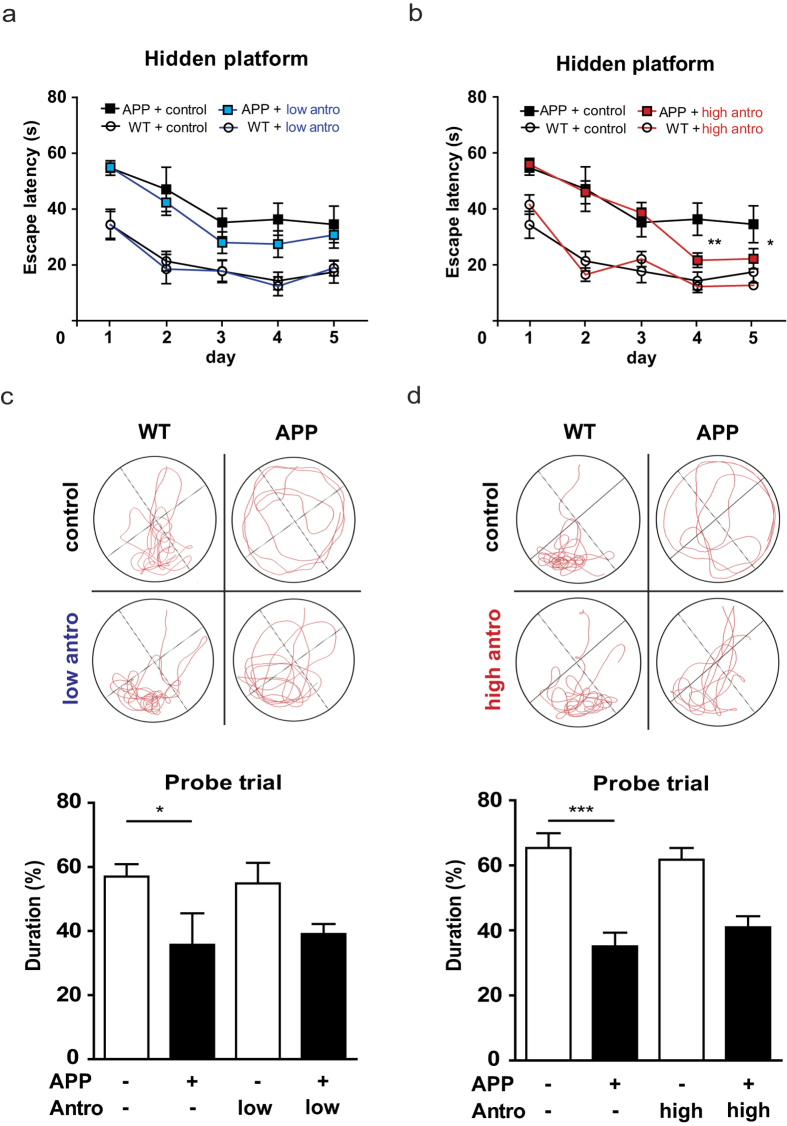
Antroquinonol improved the spatial learning and memory deficits in *APP* mice. In the Morris water maze test, *APP* transgenic mice showed poorer learning and memory compared with WT mice during five consecutive days of hidden platform training (**a**,**b**) or probe trail (**c**,**d**). (**a**,**b**) In hidden platform test, *APP* mice that consumed a low-dose antroquinonol (antro) diet (**a**) did not significanly lower escape latency, but *APP* mice that consumed a high-dose antroquinonol diet (**b**) showed a significantly lower escape latency compared with littermates given the control diet. Results were analyzed by repeated-measures ANOVA, and found an interaction between high dose antroquinonol consumption and time factors in *APP* mice cohorts. Daily performance were determined by Student’s *t* test. (**c**,**d**) In the probe trial, antroquinonol consumption did not significantly reverse the memory retention deficit. Results were analyzed by one-way ANOVA. Data are presented as mean ± SEM. Number of mice in low-dose cohort: WT + control = 6, APP + control = 4, WT + low antro = 6, APP + low antro = 8. Number of mice in high-dose cohort: WT + control = 9, WT + high antro = 7, APP + control = 8, APP + high antro = 6. *P < 0.05, **P < 0.01, ***P < 0.001.

To determine whether antroquinonol could improve the memory impairments found in *APP* mice, we used the Morris water maze test to evaluate their spatial learning and memory after 2 month of antroquinonol consumption. In hidden-platform training, a lower latency to reach the platform over five consecutive days of training indicates better memory acquisition. *APP* mice that consumed the control diet took more time to find the escape platform than WT mice (Fig. 4a,b). *APP* mice that received a low-dose antroquinonol diet did not significantly lower the time to reach the platform compared to the control diet group (Fig. 4a). However, *APP* mice that received a high-dose antroquinonol diet took significantly less time than those given the control diet group to reach platform on the fourth and fifth days of hidden-platform training (Fig. 4b). In the probe trial, *APP* mice spent less time in the target zone than WTs, suggesting the deficits in memory retention. However, antroquinonol consumption did not significantly reverse this deficit (Fig. 4c,d). Therefore, our finding suggested that antroquinonol probably help with the memory acquisition but cannot significantly improve the memory retention in *APP* mice.

### Reduction in AD-like pathology in APP transgenic mice by antroquinonol

**Figure 5:**
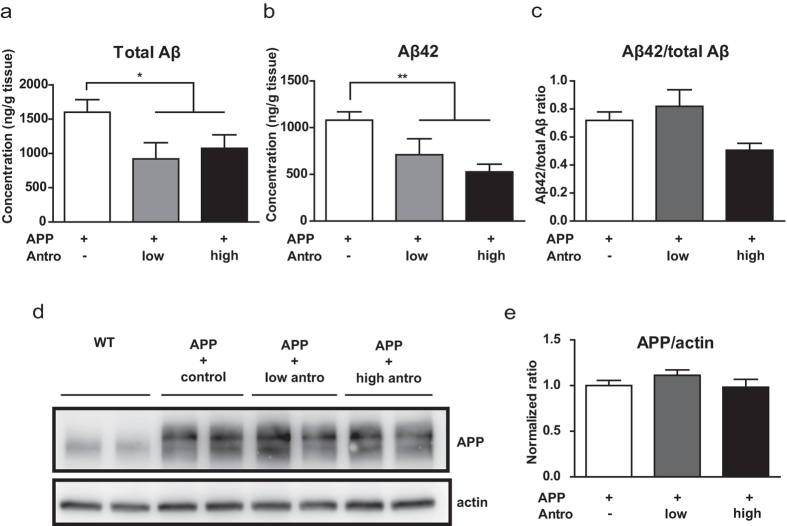
Antroquinonol reduced hippocampal Aβ but not altered APP levels in *APP* mice. Hippocampal total Aβ and Aβ42 levels in *APP* transgenic mice were measured by ELISA. (**a**–**c**) Compared to the control diet group, total Aβ (**a**) and Aβ42 (**b**) levels were significantly reduced after 2 months of antroquinonol consumption. (**c**) Aβ42/total Aβ ratio was decreased after 2 months of high antroquinonol consumption. (**d**) A representative cropped Western blot image for APP levels after antroquinonol consumption. The whole blot is shown in supplementary Fig. 2. (**e**) A quantitative analysis of the Western blot results indicated that the APP levels were unchanged after 2 months of antroquinonol consumption. Results were quantified from three independent experiments and presented as mean ± SEM. Ratio of *APP* mice given control diet was set to 1. Results were analyzed by one-way ANOVA and *t* test. Number of mice: APP + control = 11, APP + low antro = 8, and APP + high antro = 6. *P < 0.05, **P < 0.01 vs. APP + control.

**Figure 6:**
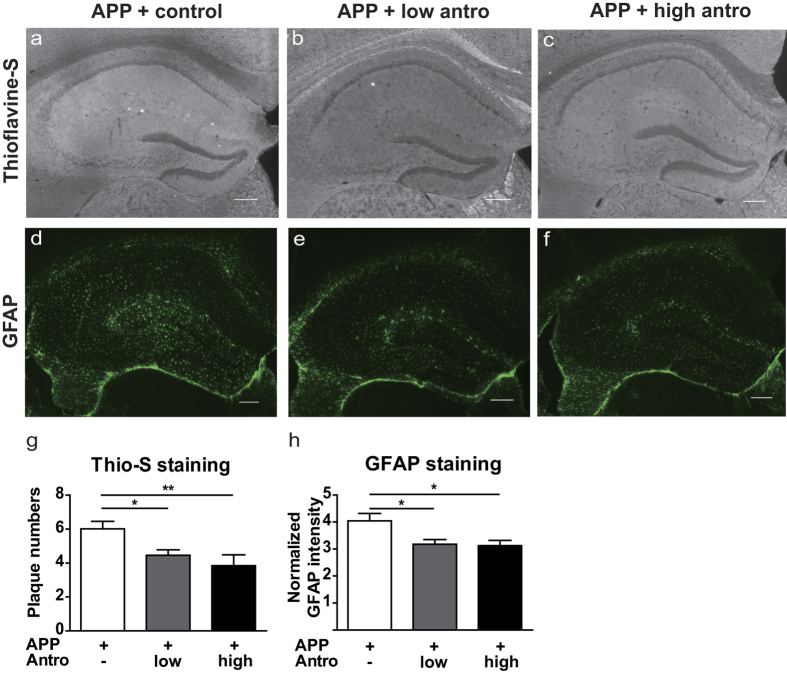
Antroquinonol reduced Aβ deposition and astrogliosis in *APP* mice. (**a**–**c**) Representative Thioflavine-S images from *APP* mice with or without antroquinonol treatment. (**d**–**f**) Representative GFAP staining images from *APP* mice with or without antroquinonol treatment. (**g**) Both low- and high-dose antroquinonol consumption reduced the number of amyloid plaques in hippocampal of *APP* mice. (**h**) Quantitative results for GFAP intensity, normalized to the size of the area measured. Both low- and high-dose antroquinonol consumption reduced GFAP expression in hippocampal of *APP* mice. Data are presented as mean ± SEM. Results were analyzed by one-way ANOVA. Number of mice: APP + control = 11, APP + low antro = 8, and APP + high antro = 6. Five to ten slices containing hippocampus were stained per mouse. *P < 0.05, **P < 0.01 vs. APP + control. Scale bar = 200 μm.

One of the histopathological hallmarks of AD is the accumulation of amyloid plaques which are mainly composed of Aβ, especially highly toxic Aβ42. Therefore, Enzyme-linked immunosorbent assays (ELISA) were performed to determine the amounts of total Aβ and Aβ42 in the hippocampus of *APP* mice treated with control or antroquinonol diet. Two months of antroquinonol consumption significantly suppressed total Aβ levels (Fig. 5a). The amount of highly pathogenic Aβ42 was significantly decreased after 2 months of high-dose antroquinonol consumption (Fig. 5b). Moroever, Aβ42 to total Aβ ratio showed a lower trend in high dose of antroquinonol treatment (Fig. 5c). However, full-length APP levels did not change in these mice (Fig. 5d,e and supplementary Fig. 2). Furthermore, the accumulation of amyloid plaques was monitored by Thioflavine-S (Thio-S) staining. Antroquinonol-treated *APP* mice had significantly lower Aβ plaque numbers than control mice (Fig. 6a–c,g).

Moreover, Aβ can trigger the neuroinflammation to produce a variety of inflammatory cytokines, thus cause neurotoxic effects in the brain. Glial fibrillary acidic protein (GFAP) expression was therefore examined as an indicator of astrogliosis after antroquinonol consumption. We found that antroquinonol significantly decreased hippocampal GFAP intensity, suggesting that antroquinonol may reduce astrocyte activation after 2 months of consumption (Fig. 6d–f,h). To sum up, these results demonstrate that antroquinonol can alleviate amyloid and inflammatory pathology in this AD mouse model.

We also examined whether the improvement of behavioral deficits is due to the rescue of neuronal or synaptic loss. Consistent with previous reports, *APP* mice had no significant amount neuron death compared with WT^29^, but showed the loss of synapse marker synaptophysin^3^. Nevertheless, antroquinonol consumption cannot reverse this loss (Supplemental Fig. 1). Therefore, the protective effect of antroquinonol may not be directly on the survival of neuron or synapse, but may due to the activating other pathways.

### Enhancement of Nrf2 and reduction of HDAC2 level in APP transgenic mice by antroquinonol

**Figure 7:**
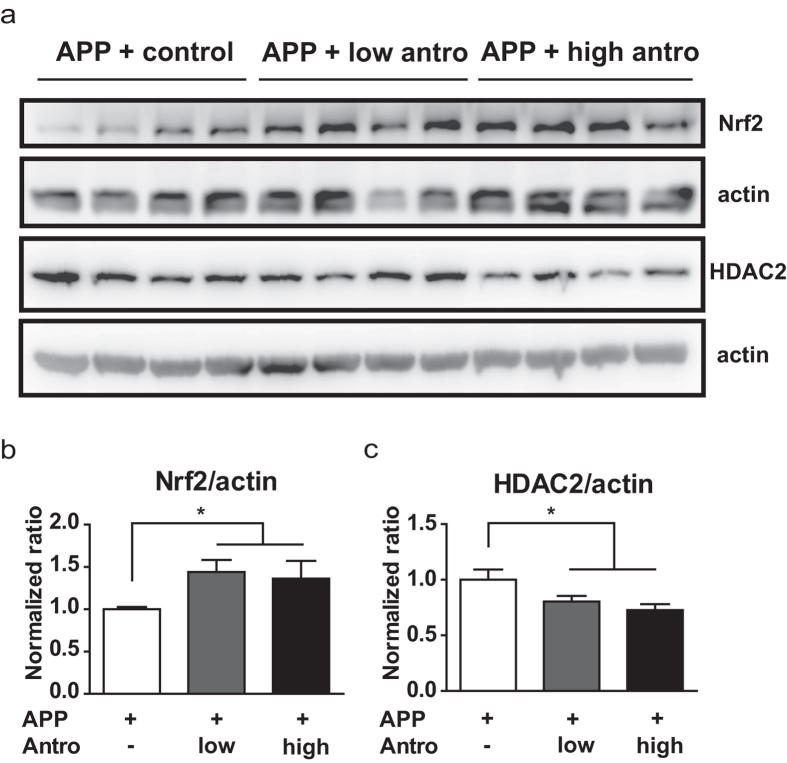
Antroquinonol increased Nrf2 and decreased HDAC2 levels in *APP* mice. (**a**) Representative cropped Western blot images showing Nrf2 and HDAC2 levels in *APP* transgenic mice. Actin was used as a loading control. The whole blot is shown in supplementary Fig. 3. After 2 months of antroquinonol consumption, (**b**) Nrf2 levels were elevated, and (**c**) HDAC2 levels were decreased. Results were averaged across four independent experiments and are presented as mean ± SEM. Results were analyzed by ANOVA and *t* test as a proportion of Nrf2 and HDAC2 levels in control-diet mice, which were set to 1. *P < 0.05 vs. APP + control.

Neuroinflammation can promote oxidative stress, and this has been shown to accelerate amyloid deposition and memory impairments in the AD mouse model^30^. Previous studies in renal system have indicated that antroquinonol is capable of reducing oxidative stress by activating the Nrf2 pathway^24^,^25^. Thus, we examined whether 2 months of antroquinonol consumption could alter the Nrf2 expression in the brain of *APP* mice. Immunoblotting results indicated that hippocampal Nrf2 was increased after administration of antroquinonol (Fig. 7a,b and Supplementary Fig. 3a). Nrf2 levels were significantly higher in the mice that consumed antroquinonol than in those given the control diet (Fig. 7b).

The activation of Nrf2 under oxidative stress could be controlled through histone deacetylase 2 (HDAC2)^31^, which negatively regulates learning and memory and is overly abundant in the hippocampus of AD mouse models^32^. We found that HDAC2 levels were decreased in *APP* mice that consumed antroquinonol (Fig. 7a,c and Supplementary Fig. 3b), suggesting that 2 months of antroquinonol consumption could have neuroprotective effects in *APP* mice via decrease of HDAC2 and increase of Nrf2 levels.

## Discussion

This study demonstrated that antroquinonol, a natural compound isolated from medicinal fungus, had no adverse effects on the animals after long-term use and was able to cross blood-brain barrier, so it could be used as a potential drug for AD. After 2 months of antroquinonol consumption, *APP* transgenic mice had an improved spatial learning and memory and fewer Aβ plaques compared to *APP* mice consumed control diet. The protective mechanism of antroquinonol may be mediated via multiple pathways. We found a reduction in astrogliosis, an upregulation of Nrf2 and a downregulation of HDAC2 levels in the brain of antroquinonol treated *APP* mice.

In this study, antroquinonol-treated *APP* mice exhibited a significant reduction in Aβ-induced reactive astrogliosis in the brain. Astrogliosis is found in AD and many other neurodegenerative diseases^10^,^11^,^33^,^34^. These over reactive astrocytes play pivotal roles in modulating neuroinflammation and oxidative stress, which are among the major factors in the pathogenesis of AD^8^,^33^. Thus, targeting neuroinflammation in the early stages is considered to be one of the approaches to treat or delay the progression of AD. Indeed, several therapeutic approaches targeting inflammatory pathways, such as non-steroidal anti-inflammatory drugs, have been tested, but most clinical trials have failed^35^. Antroquinonol exhibited potent immunomodulatory effects by reducing proinflammatory cytokine expression and serum-reactive oxygen species *in vivo*^25^,^26^. Reduced astrogliosis by Nrf2 injection showed an improvement in memory decline in the AD mouse model^10^. Therefore, the immunomodulatory effects of antroquinonol may be mediated through enhanced Nrf2 level.

Our results demonstrate that the Nrf2 level could be enhanced in *APP* mice after antroquinonol consumption, consistent with previous findings in renal inflammatory mouse^24^,^25^. Because Nrf2 levels are decreased in neurons of *APP/PS1* transgenic mice^22^ and in the AD brain^21^, antroquinonol might restore basal Nrf2 activity. Activated Nrf2 pathway could counteract Aβ-induced oxidative damage, neuronal death *in vitro*^22^,^23^, and enhance spatial learning and memory in the AD mouse model^10^. Upon exposure to reactive oxygen species, Nrf2 activates the transcription of genes involved in antioxidant protection and detoxification, including superoxide dismutases, glutathione peroxidases, peroxiredoxins, heme oxygenases and NAD(P)H:quinone oxidoreductase-1^36^. These free-radical-scavenging enzymes represent a powerful antioxidant defense mechanism to counteract damage. Various natural and synthetic Nrf2-activating compounds have been found to have beneficial effects in experimental models of neurodegeneration^36^. Therefore, antroquinonol might potentially be used as a supporting treatment for neurodegenerative diseases that involved oxidative stress and neuroinflammatory conditions.

Furthermore, we found that antroquinonol could reduce levels of HDAC2, which is known to negatively regulate memory formation and synaptic plasticity^32^. HDAC2 is the catalytic subunit of deacetylase repressor complexes, and is preferentially recruited to the promoters of neuronal-related genes^32^. HDAC2 levels are increased in multiple AD mouse models and postmortem AD patient brains via abelson murine leukemia viral oncogene homolog 1 (c-Abl) tyrosine phosphorylation^37^,^38^. Increases in HDAC2 levels and activity in AD have been linked to the worsening of neuronal and synaptic function. Treatment with HDAC inhibitors prevents the cognitive deficits and behavioral impairments in AD mice models via increasing the expression of genes involved in synaptic plasticity and memory consolidation in the hippocampus^39^. Our findings have demonstrated that the beneficial effects of antroquinonol might also mediate through the reduction of HDAC2.

We have found that 2 month of antroquinonol consumption is capable of improving learning and memory deficits, reducing brain amyloid deposition and astrogliosis, increasing Nrf2 expression, and reducing HDAC2 levels in *APP* mouse. This treatment was initiated in *APP* mice at 3 months of age, prior to the onset of AD-like pathology and functional impairments. However, it remains unknown as to whether antroquinonol could alleviate impairments after symptom onset, and whether antroquinonol is delay the onset or has prophylactic effect of AD. In conclusion, our study suggests a potential use of antroquinonol in modulating the AD pathogenesis.

## Materials and Methods

### Animals

*APP* transgenic mice (line J20), carrying the human *APP* gene with the Swedish (K670N/M671L) and Indiana (V717F) familial mutations, were used as an AD mouse model. Mice were housed in a pathogen-free facility with a light:dark cycle of 12 hours light and 12 hours dark. Food and water for mice were provided *ad libitum*. Five to six week-old male and female SD rats for toxicity study were purchased from Harlan Laboratories (Huntingdon, UK). Clinical condition, ophthalmoscopy, body weights, food consumption, hematology, urinalysis, organ weights, gross pathology, and histopathology studies were performed by a company (Aptuit, Greenwich, CT, USA). Bioavailability and tissue absorption tests were performed on BALB/c mice from Beijing Vital River Laboratory Animal Technology Company (Beijing, PR China), and were performed by WestChina-Frontier PharmaTech Co. (Chengdu, PR China). The study was approved by the Institutional Animal Care and Use Committee of National Yang-Ming University. All experimental procedures involving animals and their care were carried out in accordance with the Guide for the Care and Use of Laboratory Animals published by the United States National Institutes of Health (NIH).

### Antroquinonol administration

Antroquinonol was isolated and characterized as detailed in a previous study^40^. For the biosafety test, SD rats were administered with 10, 30, and 100 mg/kg/day antroquinonol (dissolved in olive oil), or vehicle (olive oil), orally, by gavage, daily for 4 weeks. For the bioavailability and tissue absorption tests, BALB/c mice were similarly orally dosed with 30 mg/kg for 0.5, 4, and 24 hours. For treatment of the AD mouse model, antroquinonol from Golden Technology Corporation (New Taipei City, Taiwan) was blended with rodent diet (laboratory rodent diet 5010; LabDiet, St. Louis, MO, USA) at concentrations of 0.003% and 0.015% (w/w) to produce low- and high-dose antroquinonol diets chow, respectively. Antroquinonol was replaced with olive oil to produce the control diets. Beginning at 3 months of age, mice were divided into three groups consuming low- and high-dose of antroquinonol, and control diets for 2 months. The open field, elevated plus maze, and rotarod tests were performed before and after treatment, and the Morris water-maze test was carried out after 2 months of antroquinonol consumption. Pathological and biochemical examinations were performed after all the mice had been sacrificed by intracardiac perfusion.

### Liquid chromatography-tandem mass spectrometry

The liquid chromatography-tandem mass spectrometry (LC-MS/MS) system consisted of a mass spectrometer (Quattro Ultima, Micromass Ltd, Manchester, UK), pump, and autosampler (Waters Alliance 2790 LC, Waters, MA, USA). Data were processed by MassLynx (version 4.0, Micromass Ltd, Manchester, UK). The high performance liquid chromatography conditions were as follows: Biosil column (ODS 4.6 mm × 150 mm, 5 μm; Biotic Chemical Co., Ltd, Taiwan.), mobile phase of 90% CH_3_CN + 1.0% HCOOH, and flow rate of 1.0 ml/min with a post-column split 1/10 to mass. The ionization mode involved electrospray/positive ionization, and the mass scanning mode was set to “Multiple Reaction Monitor.” The G4–parent ion *m*/*z* was 391.3, and the daughter ion *m*/*z* was 180.89. The electrospray was set to 3.2 kV, the source temperature to 80 °C, and the desolvation temperature to 400 °C. The cone, collision, and multiplier voltages were set to 15 V, 15 V and 500 V, respectively.

### Morris water maze

The water maze consisted of a water pool (122 cm in diameter) containing opaque water and a platform (14 cm in diameter) submerged 1 cm below the water surface. Hidden-platform training consisted of 10 sessions (two per day) over 5 days; each session comprised three 60 seconds trials with a 15 minutes inter-trial interval. The platform location remained constant in the hidden platform sessions, and the entry points was changed semi-randomly between days. The day after the final day of hidden-platform training, a probe trial was conducted, by removing the platform and allowing mice to explore the pool for 1 minute. The quadrant in which the platform was previous located was defined as the target quadrant, and the proportion of time (as a percentage) mice spent in the target quadrant was used to measure their memory retention. The time to reach the platform, and swim speed were recorded and analyzed with an EthoVision video tracking system (Version 3.1; Noldus, Wageningen, The Netherlands).

### Elevated plus maze

The elevated plus-shaped maze consisted of two open arms and two closed arms. Mice were placed individually in the center of the apparatus and allowed to explore for 10 minutes. The time spent, and distance travelled in each of the arms was recorded and analyzed using the EthoVision video tracking system.

### Open field

Locomotor activity in the open field was tested using the automated Flex-Field/Open field Photo-beam Activity System (Version 2.0, TRU Scan Photobeam LINC, Coulbourn Instruments, PA, USA) with a clear plastic chamber (41 × 41 × 38 cm). Two sensor frames, each consisting of a 16 × 16 photo-beam array 1.5 cm and 6 cm above the bottom of the chambers, were used to detect movements in the horizontal and vertical planes. Beam breaks in the arena were counted for 15 minutes.

### Rotarod

Mice were placed on the rod (RT-01; SINGA, Taipei, Taiwan). The rod started rotating, with an acceleration of 20 rpm/10 seconds in the first 10 seconds, and then the speed was increased from 20 rpm to 90 rpm with an acceleration of 10 rpm/10 seconds. Each speed was maintained for 5 seconds. The latency of mice to fall from the rotating rod gave a measure of their motor coordination ability.

### Enzyme-linked immunosorbent assay (ELISA)

Hippocampi from dissected brains were homogenized in 5 M guanidine/5 mM Tris buffer (pH 8.0). The samples were further diluted with chilled 0.25% casein-blocking buffer containing 0.5 M guanidine and protease inhibitor (04693116001; Roche, Basel, Switzerland). The levels of total Aβ and Aβ42 were quantified by ELISA kits (27729 and 27711; IBL, Hamburg, Germany), according to manufacturer’s protocols.

### Immunohistochemistry and Thioflavine-S staining

Paraformaldehyde-fixed brains were sectioned coronally (at 20 μm thickness), using a sliding microtome (CM1900; Lieca, Nussloch, Germany). For immunohistochemistry (IHC), slices were blocked with phosphate-buffered saline (PBS) containing 10% fetal bovine serum (FBS) and 0.5% triton X-100 for 1 hour, incubated overnight with anti-GFAP primary antibody (Z0334; Dako Cytomation, Glostrup, Denmark) and anti-synaptophysin antibody (04–1019, Millipore), at 4 °C, and then in DyLight 488-AffiniPure anti-rabbit IgG secondary antibody (111–485–003; Jackson ImmunoResearch, Westgrove, PA, USA) for 1 hour. For Thioflavine-S staining, slices were stained with 0.015% Thioflavine-S (T1892; Sigma, MO, USA) for 15 minutes at room temperature. After mounting, slides were imaged using a Zeiss fluorescence microscope (Axio Observer A1; Zeiss, Oberkochen, Germany).

### Immunoblotting

Hippocampal homogenates in 0.5 M guanidine were separated via 10% Tris-glycine polyacrylamide gel electrophoresis, transferred to nitrocellulose (66485; Pall Corp., Glen Cove, NY, USA ) or polyvinylidene fluoride (PVDF, IPVH00010; Millipore, Darmstadt, Germany) membranes, and probed with primary antibody for APP (MAB348; Millipore), Nrf2 (sc-722; Santa Cruz, TX, USA), HDAC2 (Y461; abcam, Cambridge, UK), or actin (GTX23280; GeneTex, San Antonio, TX, USA). Membranes were washed and probed with horseradish peroxidase (HRP) conjugated affinity-purified secondary antibody: goat anti-mouse IgG, goat anti-rat IgG, and goat anti-rabbit IgG (12–349, AP136P, AP132P; Millipore). Protein signals were visualized using a chemiluminescent HRP substrate detection system (WBKLS0500; Millipore) and quantified by a luminescence imaging system (LAS-4000; Fujifilm, Tokyo, Japan).

### Statistical analysis

Statistical analyses were performed with GraphPad Prism (Version 5.0; GraphPad, La Jolla, USA) or SPSS v13.0 (Version 22; IBM, New York, USA). Differences among multiple means were assessed by one-way, two-way or repeated-measures ANOVA, followed by Bonferroni’s post-hoc test. Differences between two means were assessed by paired or unpaired t test. The threshold for significance was defined as *P* < 0.05.

## Additional Information

**How to cite this article**: Chang, W.-H. *et al.* Antroquinonol Lowers Brain Amyloid-β Levels and Improves Spatial Learning and Memory in a Transgenic Mouse Model of Alzheimer's Disease. *Sci. Rep.* **5**, 15067; doi: 10.1038/srep15067 (2015).

## Supplementary Material

Supplementary Information
