# Supplementary material for: Antroquinonol Lowers Brain Amyloid-β Levels and Improves Spatial Learning and Memory in a Transgenic Mouse Model of Alzheimer’s Disease
Source: Sci Rep. 2015 Oct 15;5:15067. doi: 10.1038/srep15067 (PMC4606808; doi:10.1038/srep15067)
Supplement: Supplementary Information [file srep15067-s1.pdf]

SUPPLEMENTARY INFORMATION

Title: Antroquinonol Lowers Brain Amyloid-β Levels and Improves Spatial Learning and Memory in a Transgenic Mouse Model of Alzheimer's Disease

Authors: Wen-Han Chang, Miles C Chen, Irene H Cheng*

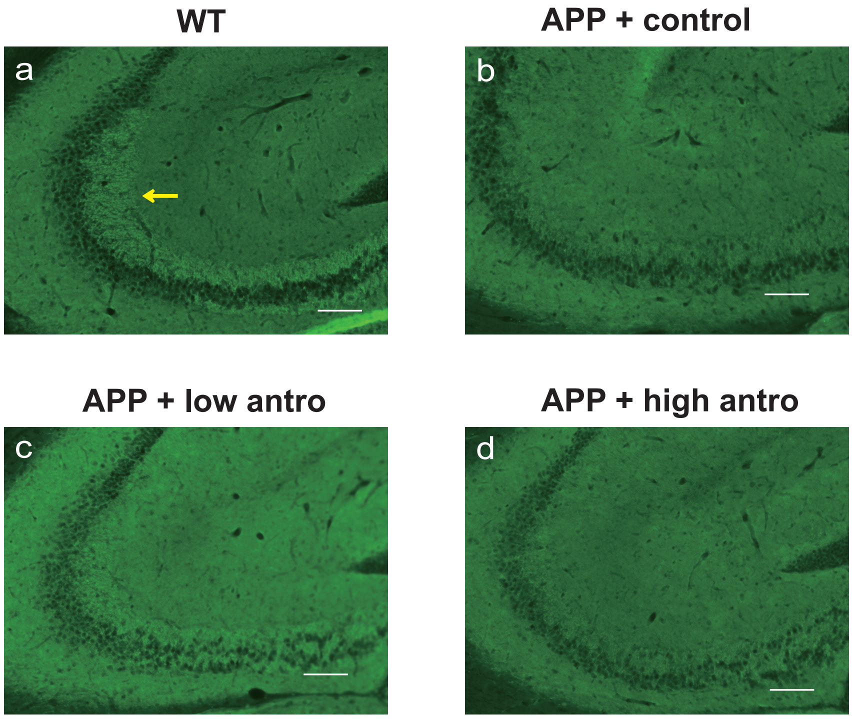

Supplementary figure 1

Representative synaptophysin images from WT and *APP* mice with or without antroquinonol treatment. (a) WT mice showed higher synaptophysin intensity at CA3 region of hippocampus (arrow) compared to (b) *APP* mice. (b-d) Antroquinonol consumption revealed a slightly improvement but did not significantly reverse the synaptophysin level in both low- and high-dose groups. Scale bar = 100 μm. Number of mice: APP + control = 5, APP + low antro = 4, APP + high antro = 4. Six to eleven slices containing hippocampus were analyzed per mouse.

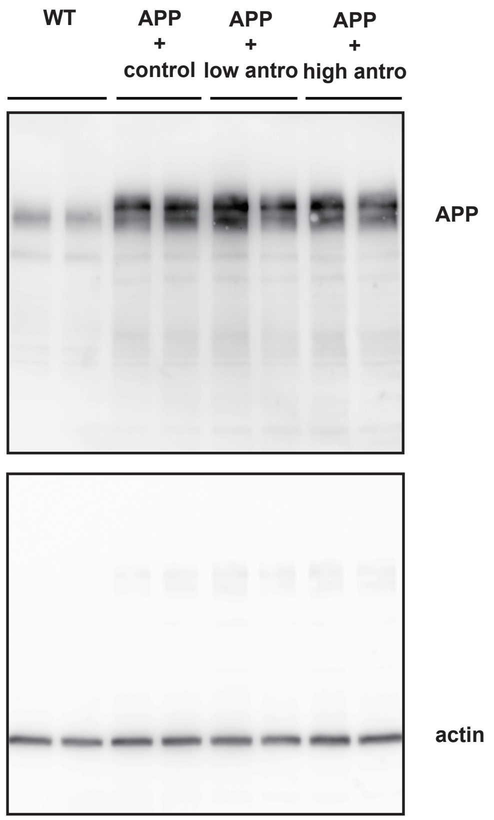

Supplementary figure 2

A representative full-length Western blot images showing APP levels in *APP* transgenic mice. APP levels were not significantly altered after 2 months of antroquinonol consumption. Actin was used as a loading control.

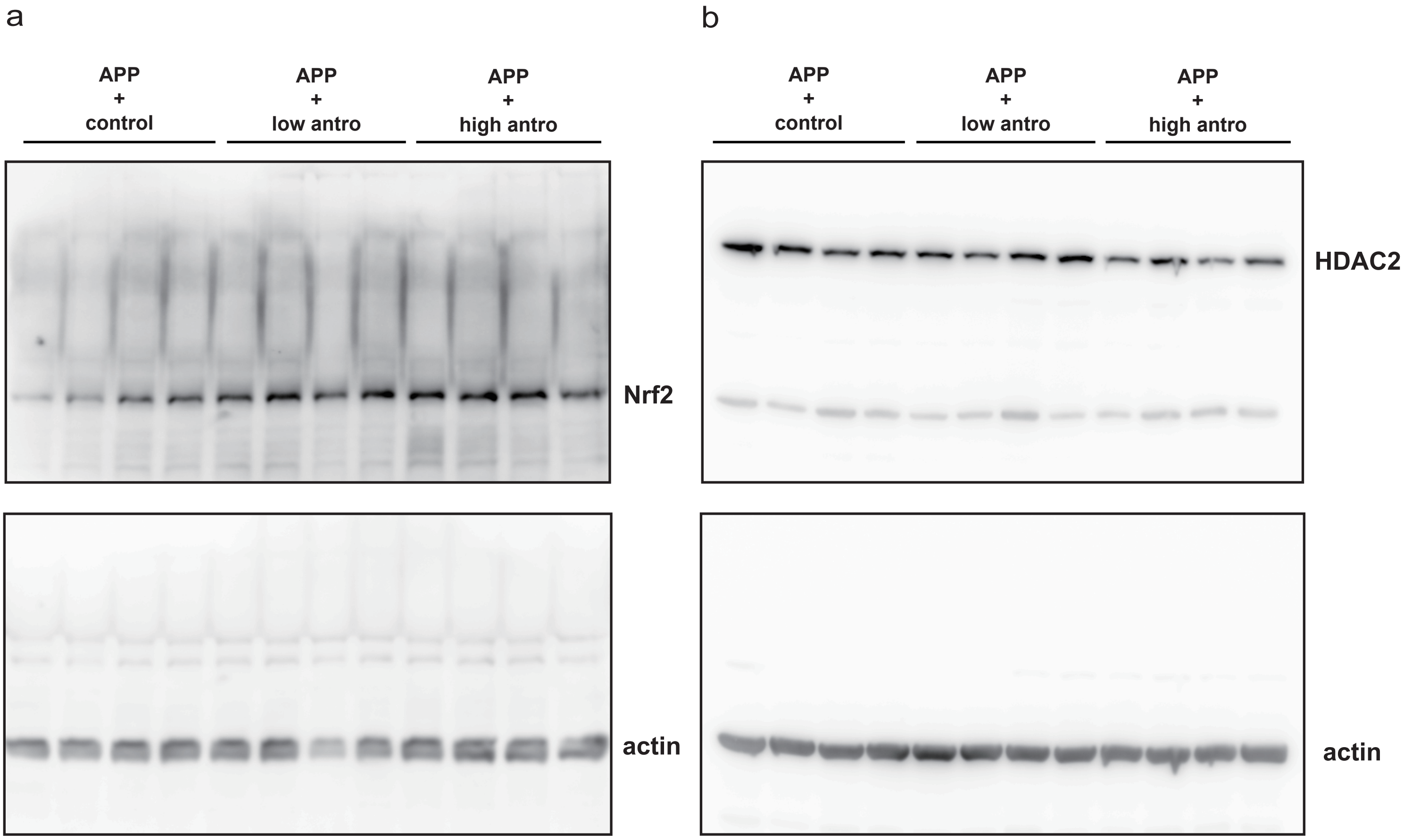

Supplementary figure 3

A representative full-length Western blot images showing Nrf2 (a) and HDAC2 (b) levels in *APP* transgenic mice. Both gels ran under the same condition as described in the Method section. Actin was used as a loading control.

Supplementary Table 1. Organ weights of antroquinonol administered SD rats.

Organs	Male (Mean ± SD)				Female (Mean ± SD)			
	0 mg/kg/day	10 mg/kg/day	30 mg/kg/day	100 mg/kg/day	0 mg/kg/day	10 mg/kg/day	30 mg/kg/day	100 mg/kg/day
**Terminal body weight (g)**	299 ± 15	293 ± 10	300 ± 14	287 ± 21	202 ± 12	202 ± 13	210 ± 14	212 ± 9
**Brain (g)**	1.76 ± 0.09	1.75 ± 0.11	1.72 ± 0.09	1.74 ± 0.06	1.68 ± 0.05	1.66 ± 0.06	1.66 ± 0.07	1.60 ± 0.07 **
**Pituitary (g)**	0.012 ± 0.002	0.011 ± 0.002	0.011 ± 0.002	0.013 ± 0.002	0.012 ± 0.002	0.011 ± 0.001	0.011 ± 0.002	0.014 ± 0.004
**Thyroid (g)**	0.015 ± 0.003	0.015 ± 0.002	0.020 ± 0.003	0.017 ± 0.006	0.018 ± 0.006	0.017 ± 0.005	0.018 ± 0.003	0.019 ± 0.005
**Thymus (g)**	0.51 ± 0.05	0.52 ± 0.08	0.73 ± 0.54	0.47 ± 0.05	0.37 ± 0.05	0.37 ± 0.06	0.37 ± 0.04	0.29 ± 0.06 **
**Heart (g)**	1.19 ± 0.1	1.19 ± 0.11	1.20 ± 0.08	1.21 ± 0.08	0.86 ± 0.05	0.91 ± 0.06	0.92 ± 0.11	0.9 ± 0.07
**Liver (g)**	13.1 ± 1.05	13.74 ± 1.03	14.04 ± 1.12	14.24 ± 1.62	7.34 ± 0.52	8.08 ± 0.93 *	8.67 ± 0.95 ***	9.75 ± 0.68 ***
**Spleen (g)**	0.74 ± 0.08	0.72 ± 0.09	0.78 ± 0.06	0.68 ± 0.09	0.57 ± 0.03	0.62 ± 0.09	0.62 ± 0.09	0.58 ± 0.08
**Adrenals (g)**	0.046 ± 0.006	0.045 ± 0.006	0.049 ± 0.006	0.056 ± 0.007 *	0.06 ± 0.008	0.063 ± 0.009	0.064 ± 0.006	0.077 ± 0.005 ***
**Kidneys (g)**	1.99 ± 0.11	2.06 ± 0.14	2.02 ± 0.12	1.85 ± 0.16 *	1.38 ± 0.1	1.43 ± 0.10	1.44 ± 0.14	1.44 ± 0.1
**Testes (g)**	4.47 ± 0.16	4.61 ± 0.15	4.63 ± 0.26	4.56 ± 0.3	0.68 ± 0.27	0.57 ± 0.09	0.63 ± 0.19	0.56 ± 0.21
**Prostate (g)**	0.38 ± 0.09	0.37 ± 0.06	0.32 ± 0.04	0.36 ± 0.08	0.085 ± 0.02	0.089 ± 0.01	0.093 ± 0.014	0.1 ± 0.009 *

* $P < 0.05$, ** $P < 0.01$, *** $P < 0.001$

Supplementary Table 2. Dose toxicity tests in antroquinonol administered SD rats.

HISTOPATHOLOGY	Males (numbers)		Females (numbers)	
	0 mg/kg/day	100 mg/kg/day	0 mg/kg/day	100 mg/kg/day
**ADRENALS**	(10)	(10)	(10)	(10)
No abnormality detected	10	9	9	4
Focal cellular change (cortex)	0	1	0	0
Cortical hypertrophy				
minimal	0	0	1	6
**HEART**	(10)	(10)	(10)	(10)
No abnormality detected	10	9	10	10
Inflammatory cell infiltrate	0	1	0	0
**KIDNEYS**	(10)	(10)	(10)	(10)
No abnormality detected	6	5	4	2
Basophilic tubules	4	5	3	3
Focal mineral deposit(s)	0	0	2	4
Nephropathy	0	0	1	2
**LARYNX**	(10)	(10)	(10)	(10)
No abnormality detected	10	10	10	9
Focal ventral pouch inflammation	0	0	0	1
**LIVER**	(10)	(10)	(10)	(10)
No abnormality detected	5	2	5	3
Inflammatory/lymphoid focus(i)	5	7	5	7
Centrilobular hepatocellular hypertrophy				
minimal	1	5	0	3
Diffuse hepatocellular hypertrophy				
slight	0	0	0	1
Clear cell focus(i)	1	0	0	0
**LUNGS**	(10)	(10)	(10)	(10)
No abnormality detected	9	9	10	9
Focal increased alveolar macrophages	0	0	0	1
Focal inflammation	1	1	0	0
**OESOPHAGUS**	(10)	(10)	(10)	(10)
No abnormality detected	9	10	10	9
Focal inflammation	1	0	0	1

**PANCREAS**	(10)	(10)	(10)	(10)
No abnormality detected	9	10	10	10
Focal acinar atrophy	1	0	0	0
**SALIVARY GLAND**	(10)	(10)	(10)	(10)
No abnormality detected	10	10	10	9
Focal PAROTID: inflammation	0	0	0	1
**SPLEEN**	(10)	(10)	(10)	(10)
No abnormality detected	8	8	7	4
Haemopoiesis	2	2	3	6
**STOMACH**	(10)	(10)	(10)	(10)
No abnormality detected	10	5	10	4
Focal ulceration with				
inflammation/necrosis in non-glandular region				
slight	0	1	0	1
Focal erosion(s) with inflammation in non-glandular				
region				
minimal	0	0	0	2
Focal inflammation in non-glandular region				
minimal	0	0	0	1
slight	0	1	0	0
Focal hyperplasia in non-glandular region				
minimal	0	0	0	1
slight	0	1	0	0
Diffuse hyperplasia in non-glandular region				
minimal	0	0	0	3
slight	0	4	0	0
**SUBMANDIBULAR LYMPH NODE**	(10)	(10)	(10)	(10)
No abnormality detected	8	5	5	3
Reactive hyperplasia	2	4	0	1
Erythrocytosis	0	1	5	6
**THYMUS**	(10)	(10)	(10)	(10)
No abnormality detected	10	10	10	7
Atrophy	0	0	0	3
**VAGINA**			(9)	(10)
No abnormality detected			9	10
Pro-oestrous morphology			2	2
**VAGINA**			(9)	(10)
Oestrous morphology			3	2

Metoestrous morphology	4	6

Supplementary Table 3. Tissue absorption of antroquinonol administered SD rats.

Group	Plasma	Fat	Spleen	Pancreas	Kidney	Uterus	Ovary	Testis	Epididymis	Urinary bladder
Post antroquinonol 0.5h	71.00	—	—	77.50	56.50	—	—	14.75	—	—
	37.25	—	—	73.50	64.00	—	—	22.85	17.35	—
	55.00	20.70	—	785.00	70.00	—	—	—	55.00	96.60
	25.60	—	10.15	180.50	99.50	98.50	—	—	—	288.00
	25.90	411.00	218.50	420.50	810.00	393.50	234.00	—	—	852.00
	15.30	—	—	20.95	50.50	35.10	—	—	—	126.00
Mean	38.34	215.85	114.33	259.66	191.75	175.70	234.00	18.80	36.18	340.65
Standard deviation	20.94	275.98	147.33	294.26	303.36	191.27	0.00	5.73	26.62	351.13
N	6	2	2	6	6	3	1	2	2	4

Group	Stomach	Intestine	Colon	Liver	Heart	Lung	Brain	Bone marrow	Skin	Skeletal muscle
Post antroquinonol 0.5h	3505.00	2505.00	187.00	38.35	66.50	198.00	17.10	—	870.00	27.00
	11550.00	7600.00	454.00	67.50	60.00	32.75	37.05	121.00	17500.00	185.00
	8350.00	19200.00	25.70	112.00	87.00	72.50	41.45	—	436.00	161.00
	14900.00	7450.00	110.50	430.50	123.50	334.50	35.30	—	2685.00	113.00
	4070.00	46000.00	61.00	355.00	51.50	202.00	68.00	—	8910.00	655.00
	3910.00	493.50	304.50	66.50	69.50	60.50	27.60	40.70	53.00	24.60
Mean	7714.17	13874.75	190.45	178.31	76.33	150.04	37.75	80.85	5075.67	194.27
Standard deviation	4737.57	17025.68	162.95	169.46	25.94	115.60	17.12	56.78	6916.98	235.30
N	6	6	6	6	6	6	6	2	6	6

Group	Plasma	Fat	Spleen	Pancreas	Kidney	Uterus	Ovary	Testis	Epididymis	Urinary bladder
Post antroquinonol 4h	19.25	—	—	—	46.65	—	—	—	119.00	—
	27.20	35.60	15.80	124.00	61.00	—	—	—	56.00	86.60
	23.70	22.80	27.15	219.50	17.75	—	—	—	59.00	114.80
	24.30	—	—	505.00	110.00	43.50	—	—	—	46.60
	16.45	27.15	12.55	94.50	32.35	69.00	—	—	—	240.00
	14.95	89.50	15.50	22.75	—	133.00	453.00	—	—	76.00
Mean	20.98	43.76	17.75	193.15	53.55	81.83	—	—	78.00	112.80
Standard deviation	4.84	30.95	6.44	188.08	35.43	46.11	—	—	35.54	75.18
N	6	4	4	5	5	3	1	0	3	5

Group	Stomach	Intestine	Colon	Liver	Heart	Lung	Brain	Bone marrow	Skin	Skeletal muscle
Post antroquinonol 4h	835.00	795.00	148.00	57.50	122.00	103.00	30.25	11.55	105.50	294.50
	21000.00	745.00	725.00	335.50	94.00	100.00	90.00	126.00	298.50	56.50
	4880.00	11000.00	9870.00	46.85	134.50	182.00	45.75	54.00	127.50	139.50
	7500.00	6550.00	2445.00	40.15	72.00	59.00	44.25	—	224.50	87.00
	12800.00	5800.00	67.50	229.00	55.00	68.00	44.35	58.50	720.00	41.95
	7700.00	24800.00	645.00	128.50	77.00	4730.00	267.50	—	950.00	88.50
Mean	9119.17	8281.67	2316.75	139.58	92.42	873.67	87.02	62.51	404.33	117.99
Standard deviation	7013.36	8969.43	3798.92	119.77	30.67	1889.71	90.73	47.32	348.39	92.75
N	6	6	6	6	6	6	6	4	6	6

Group	Plasma	Fat	Spleen	Pancreas	Kidney	Uterus	Ovary	Testis	Epididymis	Urinary bladder
Post antroquinonol 24h	—	—	—	—	—	—	—	—	27.10	—
	—	—	—	—	—	—	—	—	10.20	—
	—	—	—	18.45	—	—	—	—	—	—
	—	—	—	—	10.10	—	—	—	—	—
	—	—	—	—	—	—	—	—	—	—
	—	—	—	—	—	—	—	—	—	—
Mean	—	—	—	—	—	—	—	—	18.65	—
Standard deviation	—	—	—	—	—	—	—	—	11.95	—
N	0	0	0	1	1	0	0	0	2	0

Group	Stomach	Intestine	Colon	Liver	Heart	Lung	Brain	Bone marrow	Skin	Skeletal muscle
Post antroquinonol 24h	—	26.80	—	—	—	—	—	—	—	—
	—	—	—	—	—	—	—	—	—	—
	—	20.35	—	—	—	—	—	—	22.80	—
	835.00	63.00	—	10.10	14.80	—	—	—	38.85	23.35
	575.00	16.95	—	12.80	11.55	—	—	—	63.50	14.60
	165.00	—	—	—	—	—	—	—	11.85	—
Mean	525.00	31.78	—	11.45	13.18	—	—	—	34.25	18.98
Standard deviation	337.79	21.21	—	1.91	2.30	—	—	—	22.43	6.19
N	3	4	0	2	2	0	0	0	4	2
